# Rapid maternal death surveillance and response during a hepatitis E virus outbreak in Madani, Al Jazera state, Sudan

**DOI:** 10.1002/ijgo.71142

**Published:** 2026-06-16

**Authors:** Mawda Alshaarany, Benjamin Black, Gillian Burkhadt, Noor Rijnberg, Abrar Abdelsalam Zakria Sharif, Maura Daly, Gautam Harigovind, Ali Almohammed

**Affiliations:** ^1^ Emergency Response Team, Médecins Sans Frontières – Operational Centre Amsterdam Madani Sudan; ^2^ Médecins Sans Frontières – Operational Centre Amsterdam Amsterdam the Netherlands

**Keywords:** epidemic, hepatitis E virus, humanitarian response, maternal death, outbreak response, rapid maternal death surveillance and response tool, Sudan

A Rapid Maternal Death Surveillance and Response (R‐MDSR) tool was developed by Médecins Sans Frontières (MSF) to improve recognition and review of and response to maternal mortality during humanitarian crises. It was first implemented in Tigray, Ethiopia, in 2023 following a catastrophic conflict in the region, and has since been utilized in several locations in Sudan.^1^, ^2^

During the 2025 Acute Jaundice Syndrome (AJS)/Hepatitis E virus (HEV) outbreak in Madani, Al Jazera State, Sudan, a sharp increase in maternal deaths was identified among pregnant women presenting with AJS. AJS/HEV infection is associated with severe maternal outcomes, particularly in the second and third trimesters, with high reported case fatality rates.^3^, ^4^ As part of the humanitarian response to the AJS/HEV outbreak, and within the broader context of the ongoing Sudanese conflict and displacement that erupted in 2023,^5^ MSF teams initiated R‐MDSR to record and analyze maternal deaths associated with suspected or confirmed HEV infection, to inform and improve the response. Alongside the insecurity from armed conflict and displacement, there were challenges in accessing affected populations due to administrative policies and barriers. This resulted in the outbreak response being remotely managed by MSF, limiting the scope of activities. R‐MDSR was also conducted remotely, the first such use by MSF teams. Maternal deaths were identified through facility‐based surveillance and community notification mechanisms, and the details were recorded and reviewed using the R‐MDSR tool. The primary purpose of R‐MDSR during this period was surveillance and analytical review rather than full implementation of response actions.

During the outbreak period, pregnant women with suspected or confirmed HEV‐related complications represented a significant proportion of maternal admissions. The mortality rate among hospitalized pregnant women with AJS/HEV was 42% (22 maternal deaths, out of 53 pregnant women admitted with AJS/HEV). Most deaths occurred in late pregnancy and were associated with rapidly progressive liver failure. The limited availability of intensive care and high‐dependency unit support further constrained the management of severe cases. In addition, nine community maternal deaths from suspected HEV were reviewed using R‐MDSR. These highlighted gaps in awareness of AJS/HEV symptoms, early detection, and referral. These findings highlighted the disproportionate impact of HEV on maternal mortality during the outbreak and the critical need for strengthened referral and critical care capacity.

The R‐MDSR findings highlighted critical gaps in outbreak preparedness within maternal health services, including limited clinical awareness of AJS/HEV severity in pregnancy, insufficient diagnostic capacity, and weak linkage between infectious disease surveillance and sexual and reproductive health services.

Data generated through R‐MDSR informed internal operational discussions within MSF to strengthen referral pathways, prioritize pregnant women within AJS/HEV response activities, and integrate outbreak‐specific risks into maternal health programming. Further, review of maternal deaths identified more details on the geolocation of probable HEV outbreak hotspots, tracking of the epidemic's spread, and areas where pregnant women had the greatest challenges in accessing timely health care or where insecurity was most acute. To the authors' knowledge, this is the first recorded instance of the implementation of maternal death surveillance and response (MDSR) as an integral part of an outbreak intervention.

The remote surveillance and support model used in the response was a limitation to the effectiveness of R‐MDSR in this situation. Given the insecure setting and hard‐to‐reach populations, it was suspected that more pregnant women were infected with HEV but not reached or identified, and their outcomes are unknown. Additionally, the mortality observed was for pregnant women MSF teams were aware of, in the community or hospital, and there might have been a selection bias with milder cases not being alerted to teams. The MSF HEV response ended prematurely due to ongoing challenges with local authority partnerships and access to the affected area. Therefore, it is not possible to draw firm conclusions on the longer‐term effects of actions identified through the R‐MDSR.

When adapted to emergencies, R‐MDSR can generate real‐time evidence on preventable factors contributing to maternal deaths, supporting targeted interventions to respond to maternal mortality, including during HEV outbreaks. This experience further demonstrates the utility of R‐MDSR as a surveillance and learning tool in outbreak and humanitarian settings.

## AUTHOR CONTRIBUTIONS

All authors contributed to data review, analysis, interpretation of findings, and drafting and revision of the manuscript. All authors approved the final version of the manuscript and meet the ICMJE criteria for authorship.

## CONFLICT OF INTEREST STATEMENT

The authors have no conflicts of interest to declare.

## ETHICAL APPROVAL

This short communication piece has been approved by the MSF OCA Research Committee. No identifiable patient information was included. The R‐MDSR tool fed into program data, which was analyzed retrospectively.

## Data Availability

Research data are not shared.
